# Monodisperse silica nanoparticle suspension for developing latent blood fingermarks

**DOI:** 10.1080/20961790.2018.1446721

**Published:** 2018-03-27

**Authors:** Liang Meng, Yifei Ren, Zhilong Zhou, Congxiang Li, Chen Wang, Shanlin Fu

**Affiliations:** a Department of Forensic Science, Fujian Police College, Fuzhou, China;; b Centre for Forensic Science, University of Technology Sydney, Ultimo, Australia;; c College of Forensic Science, People’s Public Security University of China, Beijing, China;; d Narcotics Brigade, Xigang District Branch, Dalian Municipal Public Security Bureau, Dalian, China

**Keywords:** Forensic sciences, monodisperse silica nanoparticles, photonic crystal, latent blood fingermarks, fingermark development, dark non-porous substrate

## Abstract

Traditional fingermark developing methods are unsuitable for developing and extracting latent blood fingermarks on dark surfaces at crime scene because of their ineffectiveness or tedious operation procedures. In the present work, an effective and simple method was developed to serve this purpose using a suspension of monodisperse silica nanoparticles (MSNs). A suspension of 0.1 g/mL of MSNs was prepared by dispersing MSNs ultrasonically into an ethanol solution containing 1.0% Tween 80 and then uniformly sprayed onto the latent blood fingermarks on black plastic bags. Approximately 20 s later, ethanol was sprayed to clean the superfluous developing liquid. After the ethanol had evaporated, the latent fingermarks became visible as a result of the photonic crystal effect produced by the MSNs that had adsorbed to the fingermark ridges. The developed fingermarks were then photographed using a digital camera under a white or monospectral light source, revealing fine ridges and clear fingermark details. This novel, simple and effective method uses the photonic crystal effect of MSNs to develop latent blood fingermarks without the need for surface functionalization and conjugation to dye or fluorescently label molecules. The method can detect latent blood fingermarks that have been retained on a black plastic bag surface for at least 30 d. Given the simplicity and effectiveness of the developed method, MSNs may be a useful alternative material for use in developing latent fingermarks. Further research on the topic is warranted.

## Introduction

The development and extraction of latent fingermarks at a crime scene play a very important role in case investigations. Latent fingermarks require enhancement and colour development to become clear and visible [1–7]. There are well-established processes in place for developing latent fingermarks based on the types of surfaces and fingermarks involved. Blood fingermarks (fingermarks deposited in blood or generated by blood-stained papillary ridges) on dark surfaces pose a particular challenge [8,9]. When optical enhancement is unsatisfactory, chemical enhancement is often utilized to develop latent blood fingermarks. Chemical enhancement relies on the use of chemicals that target certain functional groups on biomolecules in the blood such as amino acids and proteins. The most widely used chemicals for chemical enhancement include protein dyes such as Amido Black, Acid Violet 17, Acid Yellow 7 (AY 7), Methyl Violet, Coomassie Blue and Hungarian Red that bind to the cationic groups of proteins and some reagents such as hydrogen peroxide, tetramethylbenzidine, 2, 2^′^-azino-di [3-ethylbenthiazolinesufonate], para-phenylenediamine, ortho-phenylenediamine and Leuco Crystal Violet that target the haem group of the haemoglobin molecules in blood [4,5]. Unfortunately, most of these chemical enhancement methods produce dark-coloured (black or dark violet) fingermarks that are not readily visible on a dark surface. AY 7 has been useful for revealing latent blood fingermarks on dark nonporous surfaces because the dye is fluorescent and can be visualized in fluorescence mode. However, when a relatively large amount of blood is present, AY 7 may be less effective because the haem group can reabsorb the emitted fluorescence [8,10–13]. The colour of the fingermark ridges developed by the hydrogen peroxide method tends to fade and disappear rapidly because the developing substance used is prone to absorbing oxygen in the air during fingermark development [4,5]. Additionally, it is difficult to control the concentration of hydrogen peroxide to achieve optimum fingermark development, which leads to swelling of ridges when the concentration is too high, or unclear ridges when the concentration is too low [4]. The lighter colour of 2, 2^′^-azino-di [3-ethylbenthiazolinesufonate] makes the chemical useful for developing blood fingermarks on dark surfaces because the green-coloured blood fingermark ridges that develop show some degree of contrast to the dark substrates in the absence of a multispectral light source [14]. However, the colour development procedure is usually tedious for developing latent blood fingermarks at a crime scene and is inconvenient for large surfaces [3,4].

In recent years, nanomaterials have become popular for latent fingermark development [15–22]. Nanomaterials can bind to amino acids, fatty acids or water-soluble substances in fingermarks by physical adsorption or chemical reaction. Based on the photoluminescence phenomenon of nanomaterials, clear images of fingermarks can be obtained [23–25]. At present, research efforts have focused largely on quantum dot nanomaterials, semiconductor nanocrystals and rare earth nanomaterials [17,18,26]. Although these nanomaterials are effective and convenient for fingermark development, they contain toxic heavy metals or fluorescent dyes that are harmful to the human body [4,27,28]. Titanium dioxide (TiO_2_) particles have been found to have an affinity for amino acids and proteins in latent blood fingermarks and to form light white-coloured deposits on the fingermark ridges visible to the human eye [29–32]. It has been recommended that AY 7 treatment followed by TiO_2_ treatment be used to further improve the detectability of latent blood fingermarks [9,33,34]. Very recently, Li et al. [35] successfully used NaYF_4_:Yb,Er,Gd fluorescent upconversion nanorods, a promising fluorescent label, to detect blood fingermarks on a number of substrates, and found its sensitivity and efficiency was superior to AY 7.

The use of silica-based nanomaterials for the development of latent fingermarks has gained considerable momentum [17,24,27,36–38]. Silica plays an important role in new modern materials and composite nanomaterials owing to its unique properties such as optical transparency, chemical inertness, small particle size and large surface area, high surface absorbability, favourable dispersibility and biological compatibility [39–42]. Silica materials often require initial surface functionalization with hydrophobic and/or hydrophilic groups [37,38] for better affinity followed by conjugation with dyes or fluorescent labels, including some toxic heavy metals [27,36,43] for effective fingermark visualization. Theaker et al. [37] investigated the use of hydrophobic silica nanoparticles as well as rhodamine 6G among other dyes. Moret et al. [17] synthesized silicon oxide nanoparticles functionalized by carboxyethylsilanetriol sodium salt and 3-(triethoxysilyl)-propylsuccinic anhydride for detecting natural fingermarks. These particles were shown to detect natural fingermarks on several nonporous substrates such as glass, plastics and stainless steel. Gao et al. [36] synthesized core-shell-structured CdTe@SiO_2_ quantum dots for improved detectability of latent fingermarks on a variety of surfaces because of their strong fluorescent emission. Improved latent fingermark detection was also observed by using silica dioxide (SiO_2_) nanocomposites doped with a fluorescent lanthanide (Eu^3+^)/sensitizer complex. Huang et al. [44] reported the synthesis of amphiphilic silica nanoparticles using 4-(chloromethyl) phenyltrichlorosilane and applied the materials to fingermarks on glass microscopic slides without conjugation with any dyes. It is important to note that the latent fingermarks tested with the silica-based nanomaterials described above are latent oil, sweat or oil-sweat mixed finger-marks. A complex synthesis process is necessary for the majority of silica-based nanomaterials. In addition, a nanomaterial suitable for improving detection of latent blood fingermarks by improving the contrast between latent blood fingermarks and a dark surfaces or removing the interference of fluorescence produced by some blood components has not been found.

Most silica nanoparticles currently used for developing latent fingermarks are amorphous in shape [37,38,45] or disordered [24,27,36,44]. It is known that monodisperse silica nanoparticles (MSNs) are highly uniform in size and can spontaneously self-assemble into ordered structures forming photonic crystals. By controlling the size of MSNs, by Bragg diffraction a photonic crystal diffractive wavelength within the visible spectrum can be created. This study aimed to use the photonic crystal formation property of MSNs to develop a simple and effective method to detect latent blood fingermarks on dark surfaces.

## Materials and methods

### Materials and chemicals

Tetraethoxysilane ([TEOS], 96%), ammonia (25%), sulphuric acid (98%), hydrogen peroxide (40%), sodium dodecyl sulphate ([SDS], 95%), Tween 20 (95%), Tween 80 (95%), cetyltrimethyl ammonium bromide ([CTAB], 99%), Triton X-100 (95%), polyvinylpyrrolidone-K30 ([PVP K-30], 95%) and 5-sulfosalicylic acid (95%) were purchased from the Sinopharm Chemical Reagent Co., Ltd (Shanghai, China). Ethanol was purchased from the TEDIA Company (Fairfield, OH, USA). All of the solvents and chemicals used were of analytical quality and were used without further purification unless indicated.

Whole blood was donated by the authors and stored in evacuated glass blood collection tubes containing 0.1 mg/mL EDTA (BD Vacutainer, Shanghai, China) at –25 °C. It was thawed to room temperature before use.

### Instrumentation

Photographic images were taken using a digital camera (Nikon D80; Nikon, Tokyo, Japan). A film ISO of 200, aperture of f/8, and exposure time of 1/32 s were used. Other equipment included a multispectral light source (Police eye F-400, Wulong Police Spectra Equipment Co., Ltd, Beijing, China) at 590, 540, 505, 490, 365 nm, and white light, a yellow-green filter (X0; Phenixoptics, Shangrao, China), a scanning electron microscope operated at 10 kV with a Quanta FEG 650 instrument (FEI, Hillsboro, OR, USA) and a Zeta Potential Analyser (PSS Nicomp-380/ZLS; Port Richey, FL, USA).

#### Synthesis of MSNs

The highly uniform silica colloidal microspheres were synthesized following the Stöber method [46]. The size of the silica particles could be tuned to the range of 150–400 nm by changing the formula. In this study, 8.74 g of TEOS and 180 mL of ethanol were mixed in a 250-mL flask and stirred with a magnetic beater in a 35 °C water bath. Into the mixture where then 10.0 mL of ammonia and 9.46 mL of deionized water were slowly added by dropper at left to react overnight. The 240-nm MSNs were obtained by centrifugation followed by rinsing three times with ethanol to remove residues. The nanoparticles were then oven-dried or redispersed into ethanol for preservation.

### Preparation of latent blood fingermark samples

To prepare the blood fingermarks instead of natural fingermarks, volunteers washed their fingers under flowing water and were then forbidden to touch anything. After the fingers dried naturally, they were pressed on absorbent cotton soaked with blood and then the fingermarks were stamped on the surface of black plastic (polypropylene) bags three times in succession. The last of the three fingermarks were used as the samples. The fingermark samples obtained were placed unsealed in a clean laboratory under ambient light for 1, 7, 14 and 30 d.

### The blood fingermark developing protocol

MSNs were ultrasonically dispersed into an ethanol solution containing 1.0% Tween 80 to prepare an MSN suspension concentration of 0.1 g/mL. The MSN suspension and the cleaning solution (ethanol) were then poured into an appropriate sprayer bottle separately. Subsequently, the MSN suspension was uniformly sprayed onto the latent blood finger-marks on the black plastic bags. Approximately 20 s later, ethanol was sprayed on the fingermarks to remove the superfluous developing liquid. After the ethanol evaporated, the latent blood fingermarks were vertically photographed and fixed using a digital camera mounted with a yellow-green filter under a white or monospectral light source. The aperture coefficient of the camera was set on f/8, and the exposure time was adjusted as needed.

## Results and discussion

### Developing mechanism

The Stöber method [46] is a proven and well-established method of preparing MSNs with an extremely consistent particle size. By precisely controlling the hydrolysis conditions of the TEOS and adjusting the reagent ratio or reaction conditions, silica nanoparticles with a narrow range of size distribution in adjustable sizes from 150 to 400 nm can be created. In colloidal solution, MSNs spontaneously form into orderly structures due to their intense self-assembly or intermolecular noncovalent interactions. If the relative deviation in particle size is less than 10%, the orderly structures appear as tightly stacked face- or body-centred cubes, which give rise to a 3D photonic crystal structure and exhibit the Bragg diffraction [47,48]. The diffraction wavelength obeys the Bragg equation: *m*λ = 2*nd*sinθ. Given a fixed material refractive index (*n*) and an order of diffraction (*m*), the diffraction wavelength (λ) is proportional to the lattice distance (*d*) or light incidence angle (θ). By controlling the size of the MSNs, a photonic crystal diffractive wavelength within the visible spectrum can be created. Macroscopically, the colour of the photonic crystal should be visible.

**Figure 1.**
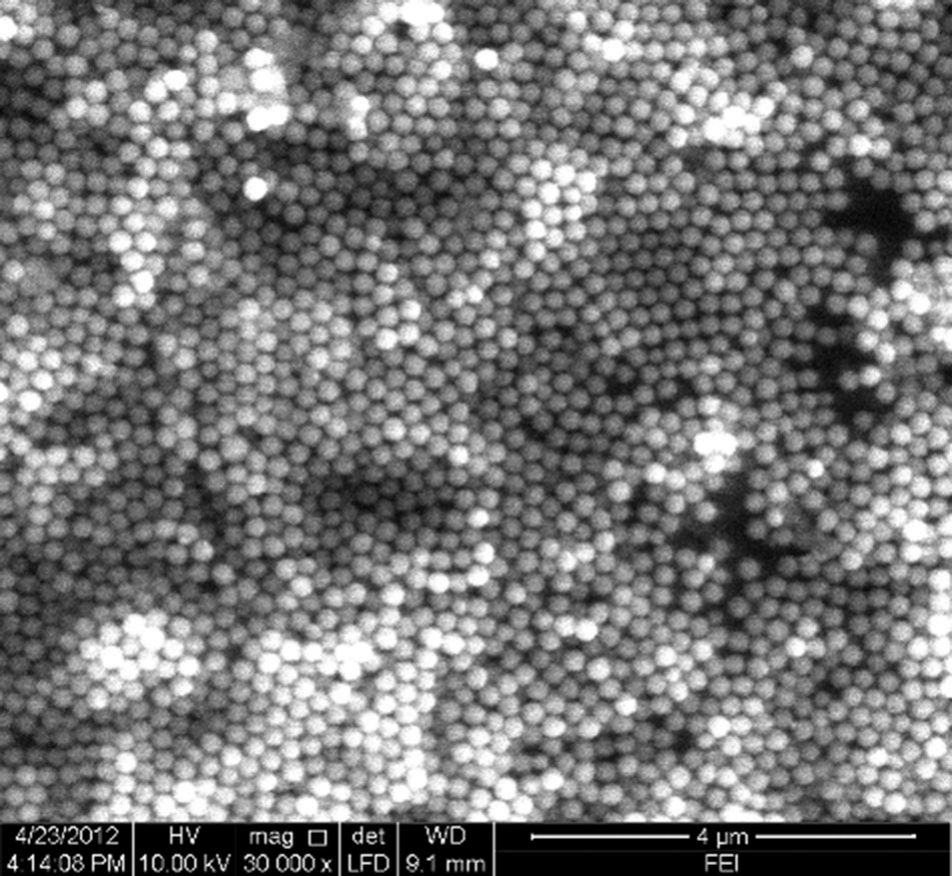
Scanning electron microscope (SEM) images of the synthesized monodisperse silica nanoparticles (MSNs).

The MSN suspension prepared in this study was dripped on a glass plate. After volatilization, the drop was observed using a scanning electron microscope. As shown in Figure 1, the nanoparticles were uniform in size. Analysis using a Nicomp-380/ZLS Zeta Potential Analyser indicated that the nanoparticles were 240 nm on average and had less than 5% dispersion variation, suggesting favourable monodispersity and self-assembly properties. By physical adsorption or intermolecular Van der Waals’ force, colloidal nanoparticles are absorbed by the residue on the ridges of the latent blood fingermarks. Subsequently, they produce photonic crystal effects by orderly layered stacking due to the self-assembly property of MSNs. When observed from vertical angles, only the raised papillary ridges show colour, and latent fingermarks are thereby developed. In addition, the fingermarks developed can display different colours by adjusting the size of the nanoparticles to enhance the contrast with the background and eliminate interference from it.

### Optimization of blood fingermark developing conditions

The blood fingermark developing protocol described in the experimental section was a result of the optimization of a number of parameters including the MSN suspension solvent, surfactant type and concentration, MSN suspension concentration and the light source used to obtain the best photographic effect. A latent blood fingermark on a black plastic bag was cut in half, with one half used for developing fingermarks during method optimization and the other half that had not undergone any treatment used for control. Each developing condition was repeated three times within 1 d. For the ease of result presentation, optimization experiments were performed by following the blood fingermark developing protocol except for the specific parameter to be optimized for which various conditions were tested.

### Selection of MSN suspension solvent

**Figure 2.**
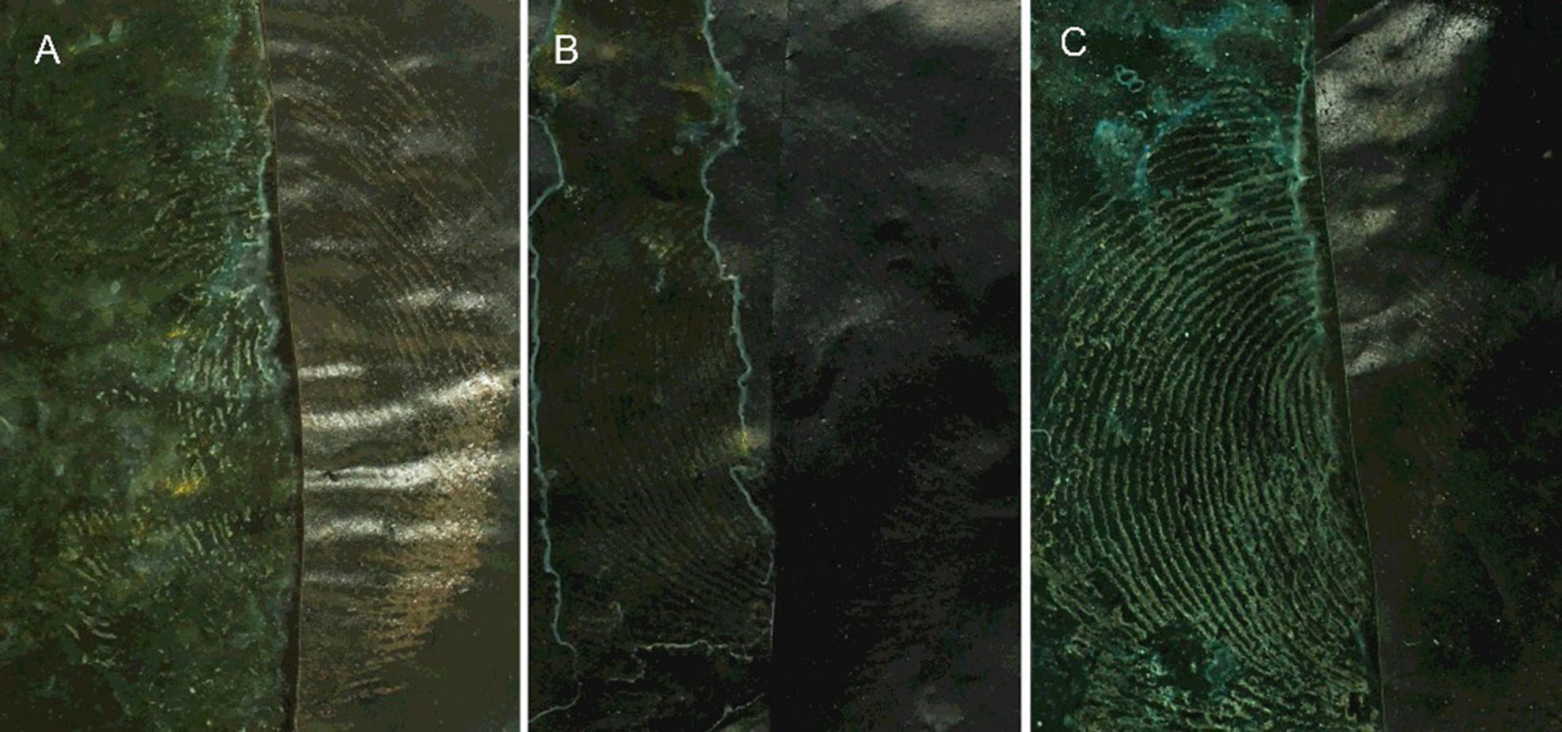
Images of the developed fingerprints by different monodisperse silica nanoparticle (MSN) suspensions prepared in water (A: fixated with 5-sulfosalicylic acid aqueous solution, B: fixated with ethanol) and ethanol, (C): the left side of the image is developed, and the right side is the original. MSN solvent was ethanol and the surfactant was 1.0% Tween 80. Camera settings: film, ISO, 200; aperture, f/8; and exposure time, 1/32 s.

The development effectiveness of MSN suspensions prepared with deionized water and with ethanol on latent blood fingermarks was investigated. Considering that spraying the water solution might damage the ridges of the blood fingermark, a fingermark fixation step was introduced when the MSN suspended in water was investigated. We found that ethanol was a better fixation solvent than 5-sulfosalicylic acid. Therefore, ethanol was chosen for fixing the fingermarks. As shown in Figure 2, the ridges of the fingermarks are displayed using the suspensions prepared with either solvent. However, the ridges were unclear when water was used as the suspension solvent, indicating that the suspension prepared in ethanol possesses superior adsorption selectivity than that prepared in deionized water. The reason for this may be that the dynamic absorption competition between water molecules and surfactant for MSNs leads to charges on the MSN surface that reduce the ability of the MSNs to be absorbed by the ridges. In contrast, this phenomenon was not observed when MSNs were uniformly suspended in the ethanol solution. Moreover, because an ethanol solution was used, there was no need to fix the blood fingermarks using ethanol beforehand.

### Selection of surfactant

**Figure 3.**
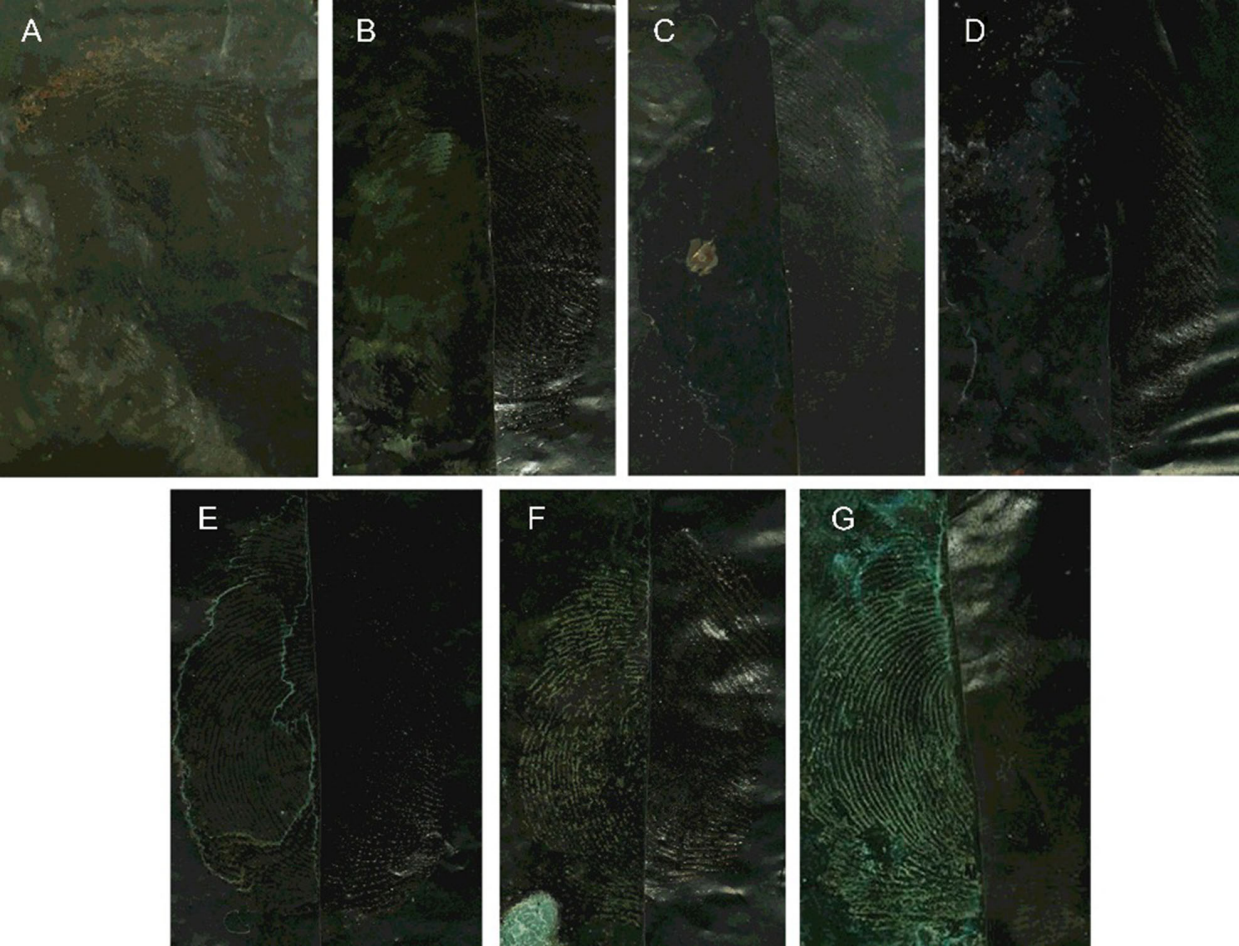
Effect of surfactant for the developing. Monodisperse silica nanoparticle (MSN) suspension concentration: 0.1 g/mL; solvent: ethanol; surfactant: (A) none, (B) CTAB, (C) SDS, (D) Triton X-100, (E) PVP-K30, (F) Tween 20 and (G) Tween 80 of 1.0%, respectively. The left side of the image is developed, and the right side is the original. Camera settings: film, ISO, 200; aperture, f/8; and exposure time, 1/32 s.

The surfactants in the suspension may enhance the binding ability of the MSNs and the residual blood substances in fingermarks. Latent blood fingermarks were separately developed using the MSN suspensions without surfactant and with six surfactants, including SDS, Tween 20, Tween 80, CTAB, PVP-K30 and Triton X-100. As shown in Figure 3, the latent blood finger-mark was almost undeveloped using the suspension without surfactant. The suspensions with the cationic surfactant CTAB or anionic surfactant SDS also resulted in unsatisfactory development. The developed ridges were blurred due to the poor absorption selectivity. This is probably because, as the charged surfactant is absorbed on the MSNs, the MSNs repel each other because they carry the same charge, making them incapable of self-assembly and ordered stacking. However, the nonionic surfactants PVP-K30, Triton X-100 and Tween 20 also failed to improve the development effectiveness. In contrast, the suspension containing Tween 80 resulted in the optimal development effectiveness. Tween 80 was chosen for further investigation although its mechanism of action remains unknown.

### The concentration of surfactant Tween 80

**Figure 4.**
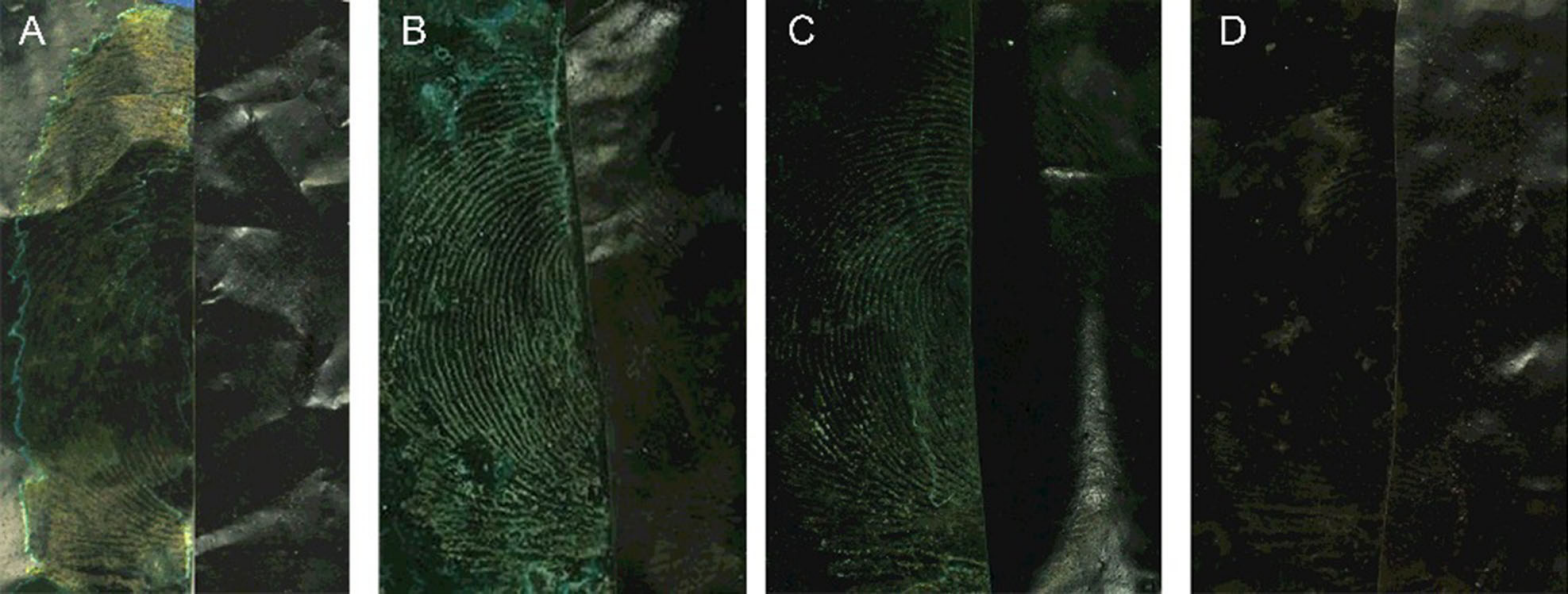
Effect of the concentration of surfactant for the developing. Monodisperse silica nanoparticle (MSN) suspension concentration: 0.1 g/mL; (A) 0.4%, (B) 1.0%, (C) 1.2%, (D) 1.5%. The left side of the image is developed, and the right side is the original. Camera settings: film, ISO, 200; aperture, f/8; and exposure time, 1/32 s.

High levels of surfactant in the suspensions may dissolve the residual blood substances and damage the fingermarks. Low levels of surfactant in the suspensions may cause ineffective binding of the MSNs to the blood fingermarks. The effectiveness of developing in suspensions containing Tween 80 at concentrations of 0.4%, 1.0%, 1.2% and 1.5% were investigated. Figure 4 shows that the developing effectiveness was enhanced as the surfactant concentration increased to 1.0% and then significantly declined at higher concentrations. Therefore, a 1.0% concentration of surfactant was chosen.

### The concentration of MSN suspension

**Figure 5.**
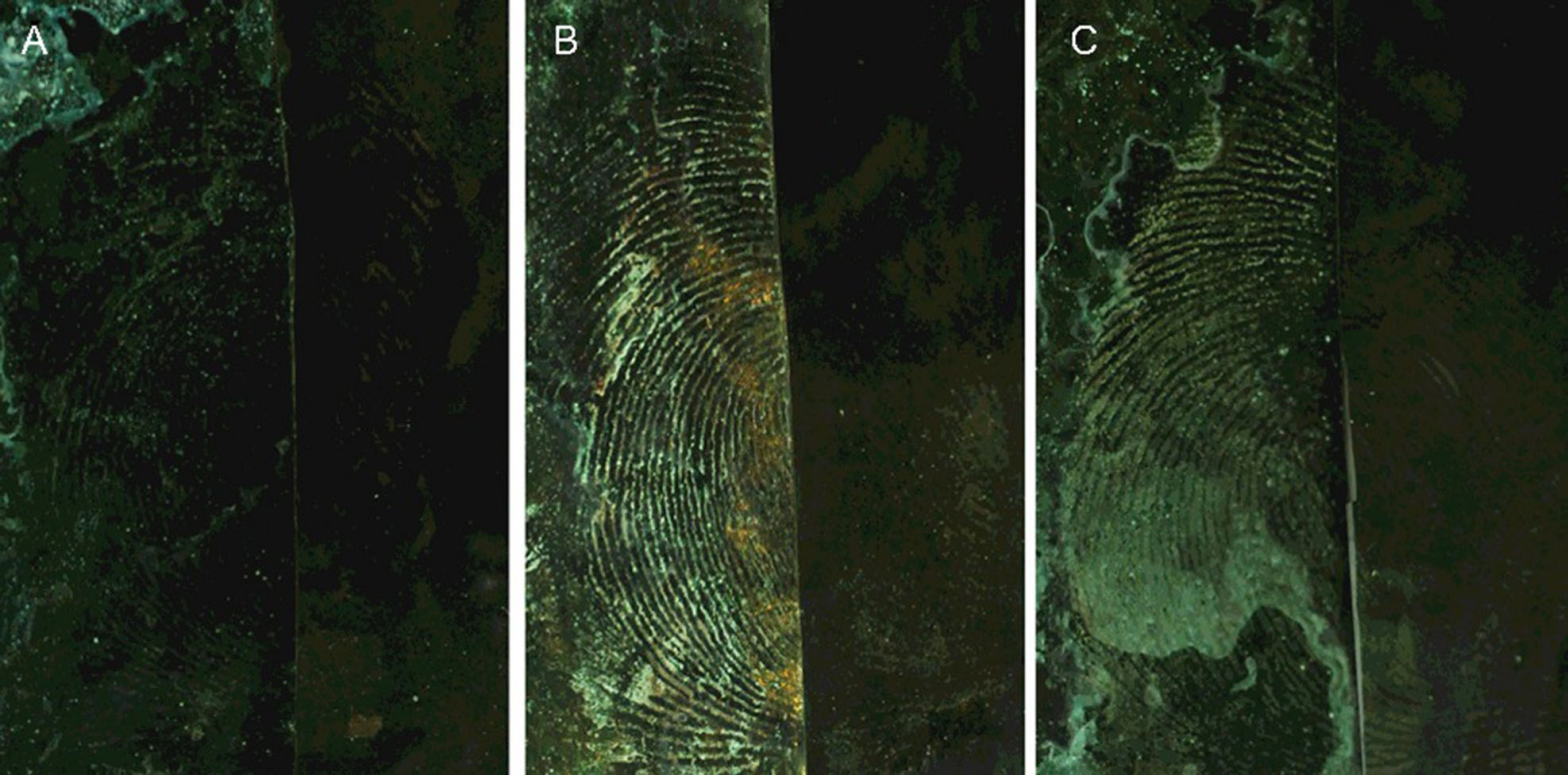
Effect of the concentration of the monodisperse silica nanoparticle (MSN) suspension on blood fingermark development. MSN suspension concentration: (A) 0.05, (B) 0.1 and (C) 0.15 g/mL. The left side of the image is developed, and the right side is the original. MSN suspension solvent was ethanol and the surfactant was 1.0% Tween 80. Camera settings: film, ISO, 200; aperture, f/8; and exposure time, 1/32 s.

Using the correct MSN suspension concentration is important to produce clear fingermark ridges. If the concentration is too low, incomplete binding of the MSNs to the fingermark ridges will occur, leading to poor fingermark development. If the concentration is too high, the MSNs can be absorbed by both the ridges and the background, resulting in a low contrast between the fingermarks and the background. The effects of developing with MSN suspension concentrations of 0.05, 0.1 and 0.15 g/mL were investigated. As the concentration of MSN suspension increased from 0.05 to 0.1 g/mL, the developing effectiveness gradually improved but then weakened as the concentration increased further (Figure 5). Therefore, the concentration of MSN suspension used was 0.1 g/mL.

### Comparisons of the photographing effects under different light sources

**Figure 6.**
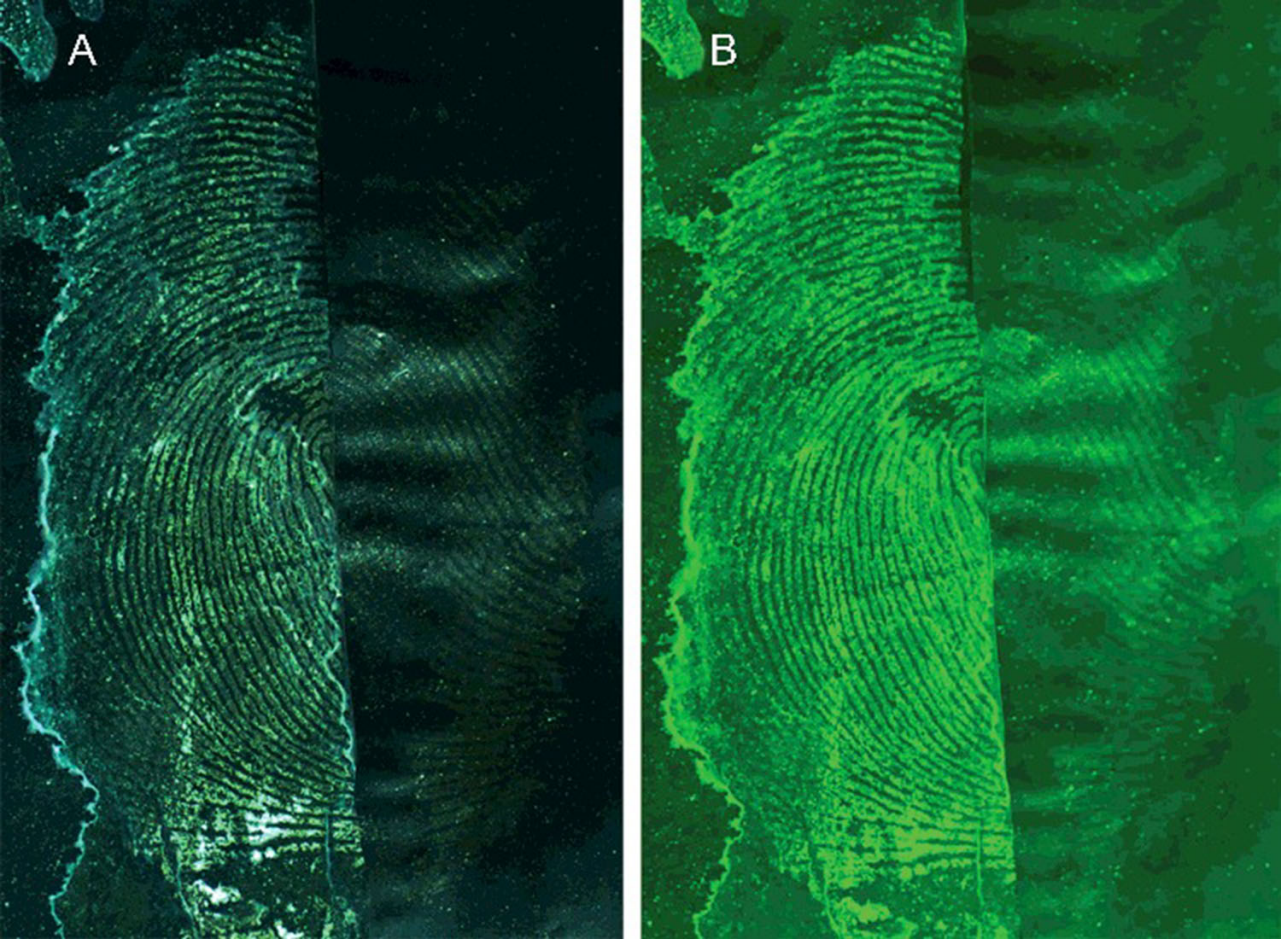
Comparison of the photographic effects under different light sources. Light source: (A) white light and (B) 540 nm. The left side of the image is developed, and the right side is the original. Monodisperse silica nanoparticle (MSN) suspension solvent was ethanol and the surfactant was 1.0% Tween 80. Camera settings: film, ISO, 200; aperture, f/8; and exposure time, 1/32 s.

The MSNs used in this study had a particle size of 240 nm and the 3D photonic crystals they assembled diffracted green light approximately 540 nm. On this basis, the photographic effects were compared under white light and 540 nm light. As seen in Figure 6, the developed ridges were clearer and contrasted with the background significantly more under the 540 nm light. Thus, the sensitivity of the development method can be increased by fitting a yellow-green filter to the white light lens used. Taking advantage of the photonic crystal formation of the MSNs when absorbed by latent blood fingermarks, different sizes of MSNs can be used to create fingermarks of different colours. The appropriate MSN size to obtain a high contrast fingermark can be chosen based on the background colour. In addition, the appropriate photographic sources should be selected to intensify the developing effect.

### The effectiveness of developing for retaining fingermarks for different time periods

**Figure 7.**
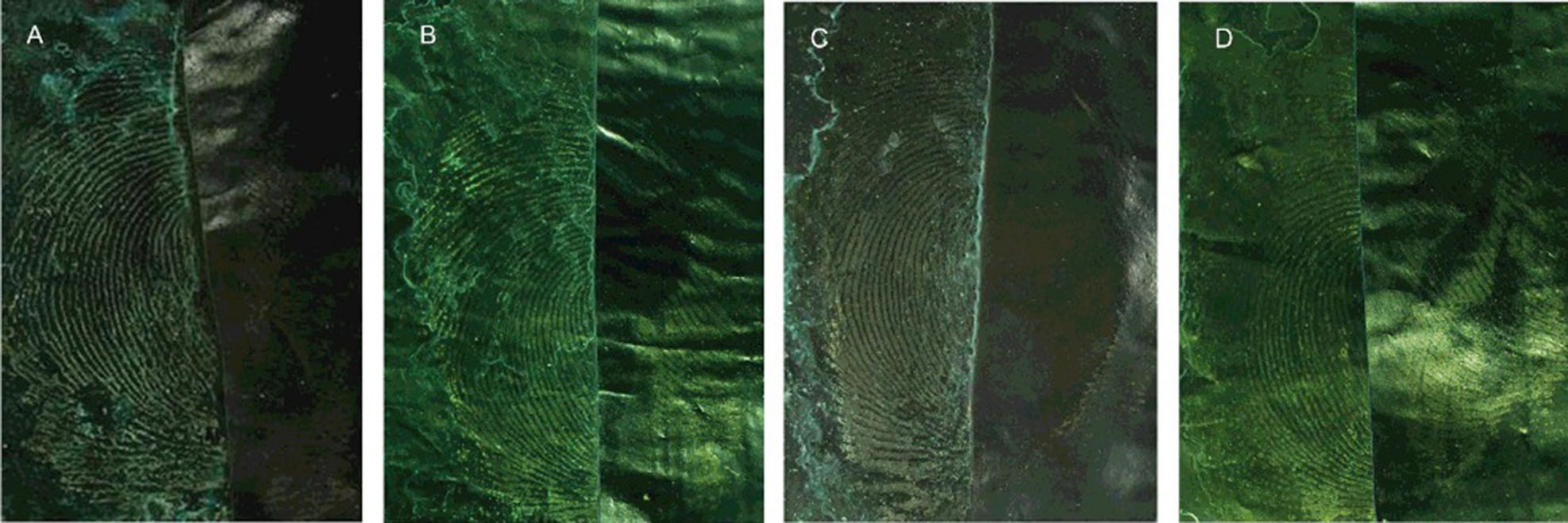
Effect of different retention times on fingermarks development. Retaining time: (A) 1, (B) 7, (C) 15 and (D) 30 d. The left side of the image is developed, and the right side is the original. Monodisperse silica nanoparticle (MSN) suspension solvent was ethanol and the surfactant was 1.0% Tween 80. Camera settings: film, ISO, 200; aperture, f/8; and exposure time, 1/32 s.

The effectiveness of developing for retaining the latent blood fingermarks on a black plastic bag for 1, 7, 15 and 30 d was investigated. Figure 7 shows that, as the retention time increased, the selective absorbability of the MSNs for latent blood fingermarks and the development effectiveness became weaker. However, the latent blood fingermarks that were retained for 30 d could still be successfully developed. The long detection window supports the view that MSNs have a strong affinity for the blood fingermarks, possibly to the proteins in them.

## Conclusion

In this study, the MSN suspension was sprayed to develop latent blood fingermarks on black nonporous plastic surfaces. This method showed high developing efficiency and clear images under white or monospectral light by simply spraying and rinsing. The ridges of the developed fingermarks were fine and smooth, and there was great contrast between the fingermarks and the background. Since the developing suspension contains ethanol, there is no need to fix the latent blood fingermarks before development. The photonic crystal effect of the MSNs eliminates the need for conjugation with dyes or fluorescent labels for latent fingermark visualization. Because of the high affinity of the MSNs for the protein molecules in the blood fingermarks, surface functionalization of the MSNs is not required. In addition, this strong affinity provides a longer window of detection with at least 30 d of blood fingermark retention time.

The photonic crystal effect produced by MSNs through self-assembly and the modulation of light waves have the potential to make the developed finger-marks different colours to enhance the contrast with the background and eliminate the background interference. By selecting the appropriate monospectral light and filter, the contrast between the fingermarks and the background could potentially be further strengthened to expand the scope of the application of this method. In future studies, focus will be placed on the developing efficiency of this method on latent blood fingermarks deposited on different porous and nonporous objects as well as on developing latent sweat or oil-sweat mixed fingermarks.
